# Molecular phylogeny and genome size evolution of the genus *Betula* (Betulaceae)

**DOI:** 10.1093/aob/mcw048

**Published:** 2016-04-11

**Authors:** Nian Wang, Hugh A. McAllister, Paul R. Bartlett, Richard J. A. Buggs

**Affiliations:** ^1^School of Biological and Chemical Sciences, Queen Mary University of London, London E1 4NS, UK,; ^2^Institute of Integrative Biology, Biosciences Building, University of Liverpool, Crown Street, Liverpool L69 7ZB, UK and; ^3^Stone Lane Gardens, Stone Farm, Chagford, Devon TQ13 8JU, UK

**Keywords:** *Betula*, convergent evolution, genome size, hybridization, ITS, phylogeny, polyploidy

## Abstract

**Background and Aims** *Betula* L. (birch) is a genus of approx. 60 species, subspecies or varieties with a wide distribution in the northern hemisphere, of ecological and economic importance. A new classification of *Betula* has recently been proposed based on morphological characters. This classification differs somewhat from previously published molecular phylogenies, which may be due to factors such as convergent evolution, hybridization, incomplete taxon sampling or misidentification of samples. While chromosome counts have been made for many species, few have had their genome size measured. The aim of this study is to produce a new phylogenetic and genome size analysis of the genus.

**Methods** Internal transcribed spacer (ITS) regions of nuclear ribosomal DNA were sequenced for 76 *Betula* samples verified by taxonomic experts, representing approx. 60 taxa, of which approx. 24 taxa have not been included in previous phylogenetic analyses. A further 49 samples from other collections were also sequenced, and 108 ITS sequences were downloaded from GenBank. Phylogenetic trees were built for these sequences. The genome sizes of 103 accessions representing nearly all described species were estimated using flow cytometry.

**Key Results** As expected for a gene tree of a genus where hybridization and allopolyploidy occur, the ITS tree shows clustering, but not resolved monophyly, for the morphological subgenera recently proposed. Most sections show some clustering, but species of the dwarf section *Apterocaryon* are unusually scattered. *Betula corylifolia* (subgenus *Nipponobetula*) unexpectedly clusters with species of subgenus *Aspera*. Unexpected placements are also found for *B. maximowicziana*, *B. bomiensis*, *B. nigra* and *B. grossa*. Biogeographical disjunctions were found within *Betula* between Europe and North America, and also disjunctions between North-east and South-west Asia. The 2C-values for *Betula* ranged from 0·88 to 5·33 pg, and polyploids are scattered widely throughout the ITS phylogeny. Species with large genomes tend to have narrow ranges.

**Conclusions** *Betula grossa* may have formed via allopolyploidization between parents in subgenus *Betula* and subgenus *Aspera. Betula bomiensis* may also be a wide allopolyploid. *Betula corylifolia* may be a parental species of allopolyploids in the subsection *Chinenses*. Placements of *B. maximowicziana*, *B. michauxii* and *B. nigra* need further investigation. This analysis, in line with previous studies, suggests that section *Apterocaryon* is not monophyletic and thus dwarfism has evolved repeatedly in different lineages of *Betula*. Polyploidization has occurred many times independently in the evolution of *Betula*.

## INTRODUCTION

Phylogenetic trees based on individual genes (gene trees) provide useful data for systematics even though the evolutionary history of a particular gene is not necessarily the same as the history of other parts of the genome, or the species (Nichols, 2001). When gene trees contradict classifications based on morphological characters, two broad categories of factors can underlie this discordance. First, a gene tree may be discordant with the species tree due to the effects of hybridization, gene duplication, polyploidy and incomplete lineage sorting (Tate and Simpson, 2003; Koonin, 2005; Degnan and Rosenberg, 2009). Secondly, morphological similarities may give a misleading phylogenetic signal due to convergence (Day *et al.*, 2014). In addition, specimens may be occasionally misidentified (Wiens, 2004), and insufficient sampling can be a problem when interpreting phylogenetic relationships (Pick *et al.*, 2010). Phylogenetic analysis of *Betula* L. (Betulaceae) is likely to be subject to these problems as *Betula* species are reported to hybridize frequently, include a number of polyploids and encompass several species that are similar morphologically (Ashburner and McAllister, 2013).

*Betula*, a genus of trees and shrubs, occupies a broad latitudinal range in the northern hemisphere, from the sub-tropics to the arctic, populating various habitats, including bogs, highlands, tundra and forests. Species of this genus occur in natural landscapes and play important roles in horticulture and forestry (Ashburner and McAllister, 2013). Although several *Betula* species have wide ranges, some have narrow ranges and are evaluated as endangered in the IUCN Red List (Ashburner and McAllister, 2013; Shaw *et al.*, 2014). The estimated species number within the genus ranges from 30 to 120 (Furlow, 1990; Koropachinskii, 2013), and new species have been described recently (Zeng *et al.*, 2008; McAllister and Rushforth, 2011; Zeng *et al.*, 2014).

The taxonomy of this genus is difficult and controversial, and several classifications have been proposed (Regel, 1865; Winkler, 1904; De Jong, 1993; Skvortsov, 2002). Regel (1865) divided it into subgenus *Alnaster* and subgenus *Eubetula*, with the former having the single section *Acuminatae* and the latter consisting of six sections (*Albae*, *Costatae*, *Dahuricae*, *Fruticosae*, *Lentae* and *Nanae*). Winkler (1904) lowered the two subgenera proposed by Regel (1865) to two sections and merged section *Dahuricae* and section *Fruticosae* of Regel (1865) into subsection *Albae*, and placed section *Lentae* into subsection *Costatae*. De Jong (1993) divided the genus into five subgenera: *Betula*, *Betulaster*, *Betulenta*, *Chamaebetula* and *Neurobetula*. Based on previous publications and specimens collected from northern Asia, Skvortsov (2002) proposed a classification of four subgenera and eight sections, namely *Asperae* (sections *Asperae*, *Chinenses* and *Lentae*), *Betula* (sections *Acuminatae*, *Apterocaryon*, *Betula*, *Costatae* and *Dahuricae*), *Nipponobetula* and *Sinobetula*. More recently, in a monograph of *Betula* (Ashburner and McAllister, 2013), a classification into four subgenera and eight sections was proposed. These subgenera are: *Acuminata* (section *Acuminatae*), *Aspera* (sections *Asperae* and *Lentae*), *Betula* (sections *Apterocaryon*, *Betula*, *Costatae* and *Dahuricae*) and *Nipponobetula* (section *Nipponobetula*), with section *Asperae* being further divided into two subsections: subsection *Asperae* and subsection *Chinenses*. This classification largely agrees with the one proposed by Skvortsov (2002), but places section *Acuminatae* (subgenus *Betula*) of Skvortsov (2002) as subgenus *Acuminata* and treats sections *Asperae*, *Chinenses* and *Lentae* of Skvortsov (2002) as subsections *Asperae*, *Chinenses* and section *Lentae*, respectively. Subgenus *Sinobetula* is not included in this recent classification since the sole species included was proposed based only on a single specimen (Skvortsov, 2002), which is considered to belong to subsection *Asperae* (Ashburner and McAllister, 2013).

Several molecular phylogenies have been published for the family Betulaceae (Bousquet *et al.*, 1992; Chen *et al.*, 1999; Forest *et al.*, 2005; Grimm and Renner, 2013) and for its constituent genera: *Alnus* (Navarro *et al.*, 2003), *Corylus* (Erdogan and Mehlenbacher, 2000; Forest and Bruneau, 2000; Whitcher and Wen, 2001), *Carpinus* (Yoo and Wen, 2002) and *Betula* (see references above). It is generally agreed that genus *Betula* is sister to *Alnus*, and the remaining four genera (*Carpinus*, *Corylus*, *Ostryopsis* and *Ostrya*) form another group (Bousquet *et al.*, 1992; Chen *et al.*, 1999). Within *Betula*, current understanding of phylogenetic relationships is based primarily on five studies with only a sub-set of currently identified species sampled in each study (Järvinen *et al.*, 2004; Li *et al.*, 2005; Nagamitsu *et al.*, 2006; Li *et al.*, 2007; Schenk *et al.*, 2008). To our knowledge, approx. 24 taxa were not included in any previous phylogenetic studies, some because they have been recently discovered or are of limited distribution, including *B. ashburneri*, *B. bomiensis*, *B. hainanensis* and *B. murrayana*. Some species placements in these phylogenies remain debated, such as the placement of *B. schmidtii* (Järvinen *et al.*, 2004; Li *et al.*, 2005), the grouping of *B. costata* and *B. alleghaniensis*, and the placement of *B. glandulosa* within section *Asperae* (Li *et al.*, 2005).

Previous comparisons of morphological and molecular classifications in *Betula* reveal that they are partially inconsistent and contradictory (Li *et al.*, 2005; Schenk *et al.*, 2008). One potential cause of this, hybridization, is known to occur frequently between *Betula* species (Dehond and Campbell, 1987; Dehond and Campbell, 1989; Nagamitsu *et al.*, 2006; Karlsdottir *et al.*, 2009; Wang *et al.*, 2014*a*) and has been shown to occur across sections and even subgenera within *Betula* (Johnsson, 1945; Dancik and Barnes, 1972; Czernicka *et al.*, 2014; Thomson *et al.*, 2015), potentially causing discordance in phylogenetic relationships.

The recent monograph of *Betula* (Ashburner and McAllister, 2013) includes determinations of the ploidy level of *Betula* species based on chromosome counts, with levels ranging from diploid to dodecaploid and counted chromosome numbers from 2*n* = 28 to 2*n* = 168. Ploidy level is an important factor in distinguishing some of the morphologically similar species in the genus, such as diploid *B. pendula* (2*n* = 2*x* = 28) and tetraploid *B. pubescens* (2*n* = 4*x* = 56); and diploid *B. ashburneri* (2*n* = 2*x* = 28) and tetraploid *B. utilis* (2*n* = 4*x* = 56). Although the ploidy level has been estimated for nearly all species of *Betula*, there are only five counts of genome size in the Plant DNA C-values Database (Bennett and Leitch, 2010), representing two diploid species, two tetraploid species and one triploid hybrid. Three of these five counts are from Anamthawat-Jónsson *et al.* (2010) where the genome size of 12 plants was measured. The genome size of another three species has been reported recently elsewhere (Bai *et al.*, 2012). Of these genome size measurements of which we are aware for *Betula*, species considered to be diploid appear to have very different genome sizes: the 2C-values of diploid species *B. populifolia*, *B. nana* and *B. nigra* were estimated to be 0·40, 0·91 and 2·90 pg, respectively (Bennett and Leitch, 2010; Bai *et al.*, 2012). Hence, there is a need for complete genome size information for the genus carried out under standard conditions with reliable identification of the specimens used.

Here, we constructed a genus-level phylogeny based on the nuclear ribosomal internal transcribed spacer (ITS) region for the genus *Betula* using only samples that have been verified by the authors of the recent monograph of the genus, Ashburner and McAllister, except in the case of four species where samples were obtained from three researchers highly familiar with them. We used the ITS region because its high level of polymorphism can help to distinguish species for phylogenetic analyses (Álvarez and Wendel, 2003) although it may suffer from complicating factors such as pseudogenes and biparental signals in recent hybrids (Razafimandimbison *et al.*, 2004). We also conducted broader analyses with samples from living collections or GenBank that have not been previously verified by the monographers. We also measured the genome size of each taxon using flow cytometry.

## MATERIALS AND METHODS

### Taxon sampling

In order to ensure a complete correspondence between the species names of Ashburner and McAllister (2013) and the taxa included in this study, we obtained species from living collections at the Stone Lane Gardens in Devon (SL hereafter) and University of Liverpool Botanic Gardens at Ness (N hereafter) since these have been collected and curated by Ashburner and McAllister. In addition, we obtained four species (*B. alnoides*, *B. delavayi*, *B. glandulosa* and *B. hainanensis*) from Jie Zeng (Institute of Tropical Forestry, Chinese Academy of Forestry), Paul Grogan (Queen’s University, Canada) and Zhikun Wu (Kunming Institute of Botany, Chinese Academy of Sciences) who have studied them over many years. We built our main phylogenetic tree using these, which we designate for the purposes of this study the ‘verified’ sample set. We then also built a phylogenetic tree including additional samples obtained from the Royal Botanic Gardens Kew, the Royal Botanic Garden Edinburgh, the Helsinki Botanic Garden, field collections (Supplementary Data Table S1) and GenBank sequences from previous published phylogenetic analyses.

**Fig. 1.**
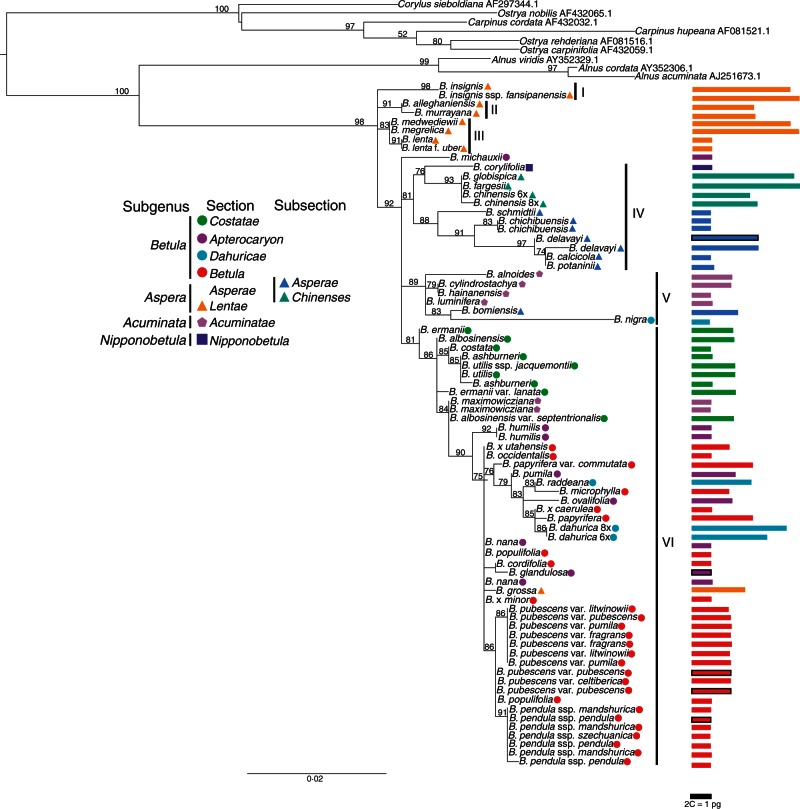
Phylogenetic tree from the maximum likelihood analysis of ‘verified’ *Betula* L. specimens using ITS sequences. Species were classified according to Ashburner and McAllister (2013). Values above branches are bootstrap percentages of ≥50 %. The bars on the right-hand side indicate genome sizes, with colours corresponding to the taxonomy. Bars with a black outline indicate a tentative genome size of the individual.

### DNA extraction, amplification and sequencing

Genomic DNA was isolated from silica-dried cambial tissue (green vascular tissue located beneath the outer bark of woody stems) or leaves following a modified 2× CTAB (cetyltrimethylammonium bromide) protocol (Wang *et al.*, 2013). The isolated DNA was assessed with 1·0 % agarose gels and measured with a Qubit 2.0 Fluorometer (Invitrogen, Life Technologies) using broad-range assay reagents. The quantified DNA was then diluted to a final concentration of 10–20 ng μL^–1^ for subsequent use. The nuclear ribosomal internal transcribed spacer (nrITS) region (ITS1, 5·8S and ITS2) was amplified using primers ITS4 (White *et al.*, 1990) and ITSLeu (Baum *et al.*, 1998). The volume of the reaction mix was 20 μL containing: 0·4 μL of AmpliTaq polymerase, 2·0 μL of 10× NH_4_ buffer (Bioline), 1·6 μL of 50 mm MgCl_2_ (Bioline), 0·5 μL of 100 mm dNTP, 0·8 μL of each primer (10 mm), 12·9 μL of ddH_2_O and 1 μL of diluted DNA (10–20 ng). The PCR was carried out using a touchdown program, consisting of an initial denaturation at 95 °C for 3 min, followed by 32 cycles of 1 min at 94 °C, 50 s at 56–52 °C, 1·5 min at 72 °C, and was ended with an extension step of 10 min at 72 °C. The PCR products were purified by binding a 0·8 vol. of Ampure beads (Beckman Coulter Inc.). The purified PCR products were diluted to approx. 20 ng μL^–1^ in ddH_2_O prior to sending them to Eurofins (Ebersberg, Germany) for sequencing.

### Phylogenetic analyses

#### ITS tree based on the ‘verified’ sample set.

Seventy-six ‘verified’ accessions representing approx. 60 *Betula* species and various subspecies, varieties and natural hybrids were Sanger sequenced. Their ITS sequences were checked for recombination in the RPD4 program (Martin *et al.*, 2015) using seven automated detection methods: Bootscanning (Salminen *et al.*, 1995); Chimaera (Posada and Crandall, 2001); GENECONV (Padidam *et al.*, 1999); MaxChi (Smith, 1992); RDP (Martin *et al.*, 2005); SiScan (Gibbs *et al.*, 2000); and 3SEQ (Boni *et al.*, 2007). No signals of recombination were detected using these methods. We downloaded ITS sequences of nine species from other genera of Betulaceae from GenBank, for use as an outgroup. In total, 85 sequences were aligned using BioEdit v 7.0.9.0 (Hall, 1999) with default parameters and the alignment edited manually where necessary. A maximum likelihood (ML) analysis was conducted in PhyML v.3.0 with the default settings (Guindon and Gascuel, 2003) and with the best-fit substitution model GTR + G selected in jModelTest 2.0 (Guindon and Gascuel, 2003; Darriba *et al.*, 2012) using the Akaike information criterion (AIC). A Bayesian inference (BI) analysis was also conducted using the program MrBayes v.3.2 (Ronquist *et al.*, 2012). Two independent runs were performed. For each run, ten million generations were completed with four chains (three heated, one cold). Trees were sampled every 1000 generations, and the first 25 % of runs were discarded as burn-in. Convergence was assessed by determining that the average standard deviation of split frequencies reached a value of <0·01. A majority-rule consensus of the remaining trees from the two runs was produced and used as the BI tree with posterior probabilities (PPs).

#### ITS tree based on all samples.

In addition to the ‘verified’ sample set, another 49 accessions were Sanger sequenced (Supplementary Data Table S1) and 99 ITS sequences of *Betula* species were retrieved from GenBank. A total of 233 sequences were aligned and analysed with ML and BI as described above. The consensus trees generated using the above methods were visualized in FigTree v.1.3.1 (http://tree.bio.ed.ac.uk/software/figtree) and edited in Adobe Illustrator CS4 (Adobe Systems).

**Fig. 2.**
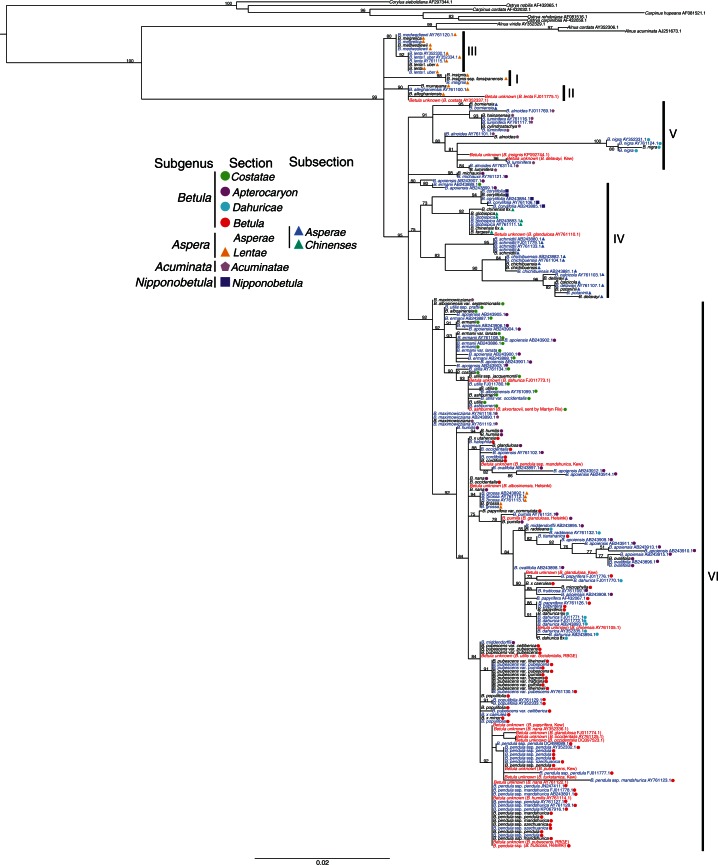
Phylogenetic tree from the maximum likelihood analysis of all *Betula* L. samples using ITS sequences. Species were classified according to Ashburner and McAllister (2013). Values above branches are bootstrap percentages of ≥50 %. Names marked in red, blue and black represent potentially misidentified accessions, potentially correctly identified accessions and ‘verified’ accessions, respectively. Included in parentheses are the original labels of potentially misidentified species and their sources; the suggested correct identification of these species is placed before the parentheses.

#### ITS tree based on diploid samples.

We also conducted phylogenetic analyses exclusively on ‘verified’ species that our C-value measurements (see below) showed to be diploid. Thirty-three *Betula* accessions were included. An ML analysis was conducted using the same parameters as described above.

### Genome size analysis

We measured the genome size of nearly all samples collected from SL and N to correlate them with ploidy levels obtained from chromosome counts (Ashburner and McAllister, 2013). Fresh leaves or cambial tissue were co-chopped with internal standards: *Oryza sativa* ‘IR36’ (Bennett and Smith, 1991), *Solanum lycopersicum* L. ‘Stupiké polní rané’ (Doležel *et al.*, 1998), *Petroselinum crispum* (Mill.) Nyman ex A.W.Hill ‘Champion Moss Curled’ (Obermayer *et al.*, 2002) and *Pisum sativum* L. ‘Minerva Maple’ (Bennett and Smith, 1991) in 1 mL of Extraction Buffer (Cystain PI absolute P, Partec GmbH, Germany) and then filtered into a tube containing 2·0 mL of Staining Solution (Cystain PI absolute P, Partec GmbH) with 12 μL of propidium iodide (PI). Samples were incubated at room temperature in the dark for approx. 30 min. Three to five replicates were analysed per sample; for each replicate, >5000 nuclei were measured using a Partec CyFlow Space flow cytometer (Partec GmbH) fitted with a 100 mW green solid-state laser (Cobolt Samba; Cobolt, Sweden). Four taxa were analysed with less than three replicates (Supplementary Data Table 1). The resulting histograms were analysed with the Flow-Max software (v.2.4, Partec GmbH).

The ranges of the species for which we measured genome size were divided into four loose categories: narrow (species occurring in a single or a few localities and tending to be endangered), medium (species occurring commonly in multiple areas), widespread (species occupying several parts of a continent) and very widespread (species spread extensively within a continent or across continents) (Supplementary Data Table S2) based on distribution information in the recent monograph of *Betula* (Ashburner and McAllister, 2013). For species in which multiple individuals were measured, the mean genome size was used for subsequent analysis. Using the average ploidy level and the mean 2C-value of each range category, statistically significant differences between categories were tested using analysis of variance (ANOVA). Tukey HSD post-hoc tests were performed at *P* < 0·05 when results of ANOVA indicated significance (α ≤ 0·05). All analyses and plots were performed in R 3.1.0 (R Develoment Core Team, 2012) and the package ‘ggplot2' (Wickham, 2009).

To investigate further the evolution of genome size in *Betula*, we calculated the monoploid genome size, 1Cx (found by dividing the 2C-value by the ploidy level of the species) (Greilhuber *et al.*, 2005), for each of the 71 verified accessions plus each accession of *B. pubescens* and *B. tianshanica* from RBGE. These 1Cx-values were grouped according to the ITS clade membership of the species; for each group, 1Cx-values were plotted against ploidy level. We also compared the homogeneity of variance for 1Cx-values among diploid (2*x*), tetraploid (4*x*), hexaploid (6*x*), and octoploid and above (8*x*–12*x*) accessions, with R package ‘lawstat’ using the modified robust Brown–Forsythe Levene-type test with 1000 bootstraps (Hui *et al.*, 2008).

## RESULTS

### The phylogeny of ‘verified’ *Betula* accessions based on ITS sequences

The aligned ITS data matrix for the ‘verified’ sample set contains 85 ITS sequences and 618 characters, of which 157 characters are variable and 111 informative. There is broad agreement between our ML (Fig. 1) and Bayesian (Supplementary Data Fig. S1) analyses; below we discuss our results based on the ML analyses as these give greater resolution. To facilitate discussion, we have labelled six main clades. Clades I, II and III consist of species of section *Lentae* (subgenus *Aspera*). *Betula alleghaniensis* is sister to *B. murrayana* whereas *B. insignis* is sister to *B. insignis* ssp. *fansipanensis*, forming clade I and II, respectively. Clade III consists of *B. lenta*, *B. megrelica* and *B. medwediewii*. Clade IV includes species of section *Asperae* and *B. corylifolia*, the single species of subgenus *Nipponobetula*, which appears to be sister to *Aspera* subsection *Chinenses*. Clade V contains all species of the subgenus *Acuminata* together with a sub-clade of *B. bomiensis* (subsection *Asperae*) and *B. nigra* (section *Dahuricae*), the latter being on a long branch. Clade VI contains all but one of the species in subgenus *Betula* plus *B. grossa* (subgenus *Aspera*, section *Lentae*) and *B. maximowicziana* (subgenus *Acuminata*). The only species of subgenus *Betula* not found in Clade VI is *B. michauxii*, which forms a polytomy with clades IV, V and VI. Within Clade VI, the various sections of subgenus *Betula* do not form unique sub-clades, though *B. costata*, *B. utilis* and *B. ashburneri* from section *Costatae* cluster together, and *B. pubescens*, *B. pendula* and their subspecies/varieties cluster together (Fig. 1). Phylogenetic relationships within the above clades are not fully resolved.

### The phylogeny of all available *Betula* ITS sequences

The aligned ITS data matrix for all accessions contains 233 ITS sequences and 622 characters, of which 188 characters are variable and 132 informative. The phylogeny of all samples (Fig. 2) reveals a similar overall topology to that of the phylogeny based only on the ‘verified’ sample set. However, 24 (16 %) of the 148 unverified samples have unexpected phylogenetic positions. Of these 24, half were downloaded from GenBank and half were sequenced from samples collected from botanic gardens. Putative *B. lenta* (GenBank accession FJ011775.1) and *B. costata* (GenBank accession AY352337.1) appear within Clade II, whereas verified accessions for these species are in Clade III and Clade VI, respectively (Fig. 2). One putative accession of *B. glandulosa* (GenBank accession AY761110.1) appeared within Clade IV, a clade of species mainly of subsection *Chinenses*, whereas another three unverified *B. glandulosa* accessions (GenBank accession FJ011774.1, RBG Kew DNA bank ID: 19950 and Helsinki Botanic Garden accession 1986-0630) are placed in Clade VI. One accession of *B. insignis* (GenBank accession KP092744) and of *B. delavayi* (RBG Kew accession 1993-3034) are unexpectedly placed within Clade V, whereas the ‘verified’ samples for these species are in Clade I and Clade IV, respectively. An accession of putative *B. dahurica* (GenBank accession FJ011773) and one of putative *B. skvortsovii* are clustered with *B. utilis* in Clade VI and one accession of *B. chinensis* (GenBank accession AY761105.1) is clustered with seven accessions of *B. dahurica* in Clade VI (Fig. 2). All the remaining 12 non-verified accessions found unexpectedly in Clade VI cluster with *B. pubescens*, *B. pendula* and their subspecies/varieties (Fig. 2).

### The phylogeny of diploid *Betula* accessions

*Betula* diploids reveal similar phylogenetic positions to when polyploids were included, with a few exceptions: *B. corylifolia* is in a polytomy with subsection *Asperae*; *B. lenta* and *B. lenta* f. *uber* are sister to species of subgenera *Betula* and *Acuminata* whereas *B. costata* clusters with subgenus *Acuminata* (Supplementary Data Fig. S2).

### Genome sizes

**Fig. 3.**
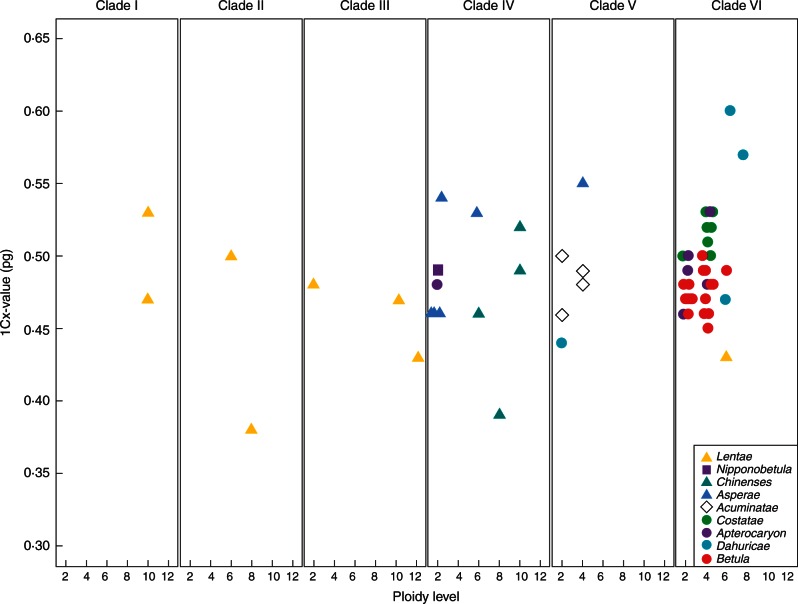
The monoploid genome size (1Cx-value) of *Betula* species and cytotypes measured from ‘verified’ samples. Ploidy levels were taken from Ashburner and McAllister (2013). Species are grouped according to the clades shown in Fig. 1.

**Fig. 4.**
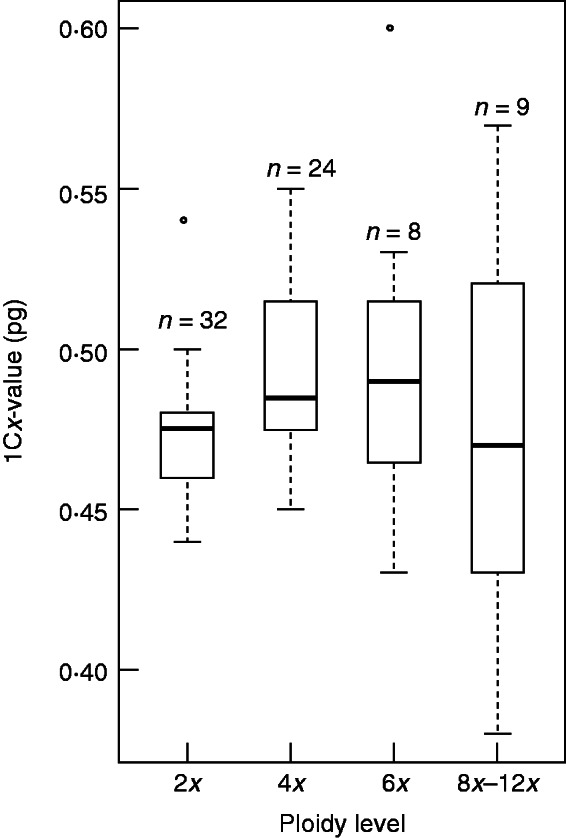
Boxplots showing *Betula* monoploid (1Cx) genome size of differing ploidy level groups: 2*x*, 4*x*, 6*x* and 8*x* and above. The number of individuals in each group is shown above the boxplot.

We found the 2C genome sizes of *Betula* species to range from 0·88 pg in *B. nigra* to 5·33 pg in *B. insignis* ssp. *fansipanensis*, thus the 1C-value ranges from 0·44 pg (430 Mbp) to 2·67 pg (2611 Mbp). We found Chinese *B. alnoides* to have a 2C genome size of 1·95 pg, indicating that it is tetraploid rather than diploid (Fig. 1; Supplementary Data Table S1). The fact that *B. alnoides* is tetraploid has been confirmed by chromosome counting and microsatellite genotyping (Hugh McAllister and Jie Zeng, pers. comm.). We found a genome size of 0·91 pg for *B. hainanensis*, indicating for the first time that this recently discovered species is diploid. If all other ploidy levels given in Ashburner and McAllister (2013) are correct, the monoploid genome size of *Betula* (1Cx-value) ranges from 371 Mbp for *B. murrayana* to 616 Mbp for *B. dahurica* (Fig. 3). The monoploid genome size is similar among all diploids except for *B. potaninii*. Variance in monoploid genome size is greater among polyploid accessions. There is a significant difference in the variance of 1Cx-values among the groups of 2*x*, 4*x*, 6*x* and 8*x*–12*x* accessions [Fig. 4, *P* < 0·05; treated pairwise, all groups are significantly non-homogenous in their variances except 4*x* and 6*x* (*P* = 0·15) and 6*x* and 8*x*–12*x* (*P* = 0·38)]. The proportion of polyploid species of this genus is approx. 0·60, if only species, subspecies/varieties and different cytotypes are included and species having synonyms are treated as one.

**Fig. 5.**
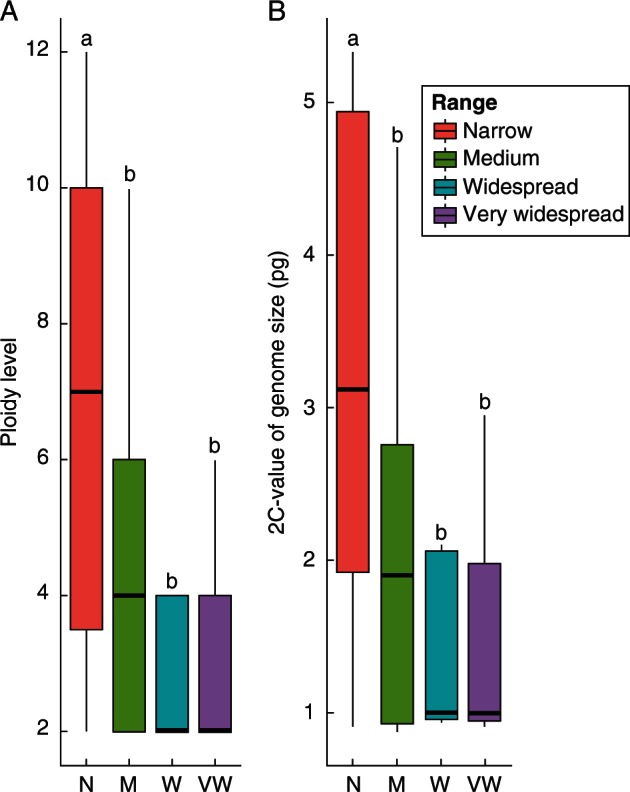
The average ploidy level (A) and the 2C value of genome size (B) among species of different ranges followed by the number of species in parentheses: narrow (12), medium (21), widespread (5) and very widespread (7), respectively. Letters a and b indicate differences at the significance level of *P* ≤ 0·05. There is no significant difference in the average ploidy level or the 2C-value of genome size if different categories share the same letter.

There is a significant difference in the average ploidy level between species with narrow ranges and species with medium, widespread and very widespread ranges (Fig. 5A, *P* < 0·05), with species with narrow ranges tending to have higher ploidy levels. There is no significant difference in the average ploidy level for species with medium, widespread and very widespread ranges (Fig. 5A, *P* > 0·05). Similar results also hold true for 2C-values (Fig. 5B).

## DISCUSSION

### Phylogenetics and taxonomy

#### Subgenus *Aspera.*

Ashburner and McAllister (2013) divided subgenus *Aspera* into two sections: section *Lentae* (from Regel 1865) and section *Asperae*. Our ITS data support this division, as the majority of species in these two sections fall into distinct ITS clades, though section *Lentae* is further subdivided into three unresolved clades. The amplified fragment length polymorphism (AFLP) data of Schenk *et al.* (2008) also agree with the division of sections *Lentae* and *Asperae*. Ashburner and McAllister (2013) further divided section *Asperae* into subsections *Chinenses* and *Asperae*, which are synonymous with section *Chinenses* and section *Asperae* of Skvortsov (2002), respectively. Our ITS data broadly support this division. Our ITS data do not support Winkler’s (1904) combination of sections *Lentae* and *Costatae* of Regel (1865) into subsection *Costatae*, nor do the data support subgenus *Neurobetula* of De Jong (1993), which consists of species from section *Asperae*, section *Costatae* and section *Dahuricae* of Ashburner and McAllister (2013). In addition, our ITS data do not support subgenus *Betulenta* of De Jong (1993) including species such as *B. lenta*, *B. lenta* f. *uber* and *B. globispica* as *B. globispica* is placed in a distinct clade (Fig. 1).

The tetraploid species *B. bomiensis*, which Ashburner and McAllister (2013) place within section *Asperae*, is clustered by ITS into a group of species of subgenus *Acuminata*, but as sister to *B. nigra* which Ashburner and McAllister (2013) place in section *Dahuricae.* As Ashburner and McAllister (2013) note, *B. bomiensis* is morphologically similar to *B. potaninii* (section *Asperae*), suggesting that this diploid species may be a parent of *B. bomiensis*. Our genome size data support this hypothesis, in that the monoploid genome size (1Cx) is unusually large for both species (0·54 pg for *B. potaninii* and 0·55 pg for *B. bomiensis*) (Fig. 3; Supplementary Data Table S2). The hypothesis that *B. bomiensis* was formed via hybridization between *B. potaninii* and a species of subgenus *Acuminata* merits further research with additional genetic loci.

Decaploid species *B. medwediewii* and dodecaploid species *B. megrelica* form a well-supported clade with diploid species *B. lenta* and *B. lenta* f. *uber* (Figs 1 and 2). This suggests that *B. lenta* or its ancestral lineage may have been a parent of these two polyploid species. The morphology of the three species also supports this hypothesis (Hugh McAllister, unpubl. res). The study of Li *et al.* (2005) found a similar result that *B. lenta* and *B. lenta* f. *uber* formed a clade with *B. medwediewii*. It has previously been suggested (Barnes and Dancik, 1985) that the octoploid species *B. murrayana* is a recent allopolyploid derivative from *B. × purpusii*, an inter-subgenus hybrid between *B. alleghaniensis* (8*x*) and *B. pumila* (4*x*). We find it to form a clade with *B. alleghaniensis* in the ITS tree, supporting this species as one of its parents (Ashburner and McAllister, 2013).

Interestingly, *B. delavayi*, a hexaploid species, clustered with the diploid species *B. calcicola* and *B. potaninii*, indicating that one of these species or their common ancestor could be a parental species of *B. delavayi*. Interestingly, both *B. potaninii* (1Cx = 0·54 pg) and *B. delavayi* (1Cx = 0·53 pg) have an unusually large monoploid genome size, which could be evidence favouring *B. potaninii* as its parental species rather than *B. calcicola* (1Cx = 0·46 pg). Further research is needed to confirm whether other species may also be potential progenitors of *B. delavayi*.

Ashburner and McAllister (2013) place the hexaploid species *B. grossa* in section *Lentae* due to clear morphological similarities, but is not clustered with species of that section by ITS sequences (Fig. 1). This is consistent with AFLP data of Schenk *et al.* (2008) and the ITS sequences of Nagamitsu *et al.* (2006). In our case, both *B. grossa* accessions are from different botanic gardens but each shows the same result (Fig. 2), making misidentification less likely. The unexpected placement of *B. grossa* into a clade of species of subgenus *Betula* may indicate that one of the progenitors of this polyploid belongs to subgenus *Betula*. It is perhaps an allopolyploid formed from hybridization with a species of section *Lentae* to which it has morphological similarity, causing McAllister and Ashburner (2013) to place it in that section. The ITS sequences from *B. grossa* may be homogenized from one parent (Nagamitsu *et al.*, 2006). This hypothesis for the parentage of *B. grossa* deserves further investigation with a larger number of genetic loci.

#### Subgenus *Nipponobetula*.

Subgenus *Nipponobetula*, which comprises the single species *B. corylifolia*, with distinctive morphology, forms a moderately supported clade (IV) with species of subgenus *Aspera* in this study, with which it shares some morphological features (Ashburner and McAllister, 2013). Our data do not support the placement of *B. corylifolia* in section *Costatae* as in Regel (1865), or subsection *Costatae* as in Winkler (1904), or subgenus *Betulenta* as in De Jong (1993). The placement of *B. corylifolia* with subgenu*s Aspera* was also indicated in two previous phylogenetic studies (Li *et al.*, 2005; Nagamitsu *et al.*, 2006). However, we note that *B. corylifolia* is found in an ITS clade within *Aspera* that is composed of the polyploid species *B. chinensis* (hexaploid and octoploid), *B. fargesii* and *B. globispica*, and this clade of four species is sister to a clade containing the diploid *Aspera* species, of subsection *Asperae*. We cannot therefore exclude the possibility that *B. corylifolia* is a parental species of allopolyploids *B. chinensis* (hexaploid and octoploid), *B. fargesii* and *B. globispica*, through hybridization with a species from section *Asperae*, and may appear nested in the subgenus *Aspera* as a result. Indeed, in phylogenetic analyses that include only diploid species, *B. corylifolia* is not nested within subgenus *Aspera*, but in a polytomy with that clade.

#### Subgenus *Acuminata*.

The subgenus *Acuminata* does not form a distinct clade in our ITS phylogenies. Four of its species appear in a clade with *B. nigra*, an outlier from subgenus *Betula*, and *B. bomiensis*, an outlier from subgenus *Aspera*. Of these four species, *B. alnoides* and *B. cylindrostachya* are tetraploid and *B. hainanensis* and *B. luminifera* are diploid species, suggesting that one or both of the two diploids or their common ancestor could be parental species of the tetraploids. A fifth species of *Acuminata*, *B. maximowicziana*, appears in the subgenus *Betula.* A close relationship of *B. maximowicziana* with species of section *Costate* (subgenus *Betula*) is also supported by AFLP markers (Schenk *et al.*, 2008) (though other species of subgenus *Acuminata* were not included in the AFLP study of Schenk *et al.*, 2008). In contrast, the low-copy nuclear gene *NIA* supports the grouping of *B. maximowicziana* with *B. alnoides*, another species of subgenus *Acuminata* (Li *et al.*, 2007), making the phylogenetic position of this species questionable. Two lines of evidence in addition to our ITS results may suggest that *B. maximowicziana* is closely related to species of subgenus *Betula*. First, a crossing experiment apparently showed that fertile hybrids can form between *B. maximowicziana* and *B. pendula* ssp. *mandshurica* (Johnsson, 1945), indicating that no post-zygotic barriers exist; however, this result has not been convincingly reproduced and we thus cannot exclude the possibility that pollen contamination could have occurred. Secondly, the autumn fruiting and much thicker male catkins of *B. maximowicziana* are distinct from other species of subgenus *Acuminata* (Ashburner and McAllister, 2013). Although the overall appearance and detailed characteristics of *B. maximowicziana* suggest a close relationship with other species of subgenus *Acuminata*, it does stand apart from them in several features, suggesting an ancient genetic contribution from another evolutionary line within the genus. If the subgenus *Acuminata* is not monophyletic, the racemose pistillate inflorescence which characterizes it is possibly due to convergent evolution.

#### Subgenus *Betula.*

The majority of the species of the subgenus *Betula* form a single clade, but the four sections of this subgenus have complex relationships in the ITS tree. Section *Costatae* shows a close relationship with section *Betula*, and section *Apterocaryon* species are intermixed with section *Betula* (Figs 1 and 2). Species of section *Betula* may have diverged from a lineage of section *Costatae* recently as the reproductive barrier between the two sections is incomplete: hybrids have been created and reported to be fertile, such as *B. pubescens* × *B. ermanii*, *B. pubescens* × *B. albosinensis* and *B. pendula* × *B. ermanii* (Johnsson, 1945). The status of section *Apterocaryon*, containing *B. michauxii* and *B. apoiensis*, *B. nana*, *B. ovalifolia*, *B. fruticosa*, *B. pumila*, *B. humilis* and *B. glandulosa*, defined by dwarf character, is not supported by the ITS tree, which indicates that the dwarf birches are heterogeneous (Figs 1 and 2). This study, together with several other studies (Li *et al.*, 2005, 2007; Schenk *et al.*, 2008), suggests that dwarfism is a convergent trait, perhaps due to adaptation to cold temperature as evidenced by the existence of bud scales (De Jong, 1993). *Betula nana* shows a closer relationship with *B. pubescens*/*B. pendula* than with *B. humilis* (Fig. 1). A similar result has been indicated by *ADH* (Järvinen *et al.*, 2004) and *NIA* (Li *et al.*, 2007). In addition, the more similar flavonoid profiles of the buds of *B. nana* and *B. pubescens* compared with those between *B. nana* and *B. humilis* (Wollenweber, 1975) suggest a closer relationship of the former pair than the latter. Surprisingly, *B. michauxii*, a species morphologically almost identical to *B. nana*, is not placed within subgenus *Betula* (Fig. 1), which is consistent with the *NIA* phylogeny (Li *et al.*, 2007). Further research is needed to decipher the phylogenetic position of *B. michauxii*.

The taxonomies of the widespread species *B. pendula* and its tetraploid relative *B. pubescens* have been particularly controversial in the past, with several subspecies or varieties of both being described and sometimes classified as independent species. Our analysis (Figs 1 and 2) supports the taxonomic treatment of these two species suggested by Ashburner and McAllister (2013), where taxa within the two species are not given species status. *Betula pubescens* is a tetraploid species; its close relationship with *B. pendula* indicates the possible involvement of *B. pendula* in its formation, as has previously been suggested (Howland *et al.*, 1995). The morphological diversity found within these species is probably due to their wide distribution ranges, with morphological variation shaped by overall climatic factors, similar to the variation found within *B. papyrifera* in North America (Pyakurel and Wang, 2013). Another factor may be hybridization and gene flow between *Betula* species in different areas of their distributions.

Within section *Costatae*, *B. costata* forms a well-supported clade with other species of section *Costatae* such as *B. utilis* based on ITS data (Fig. 1). This supports the inclusion of *B. costata* and *B. utilis* in section *Costatae* (Skvortsov, 2002; Ashburner and McAllister, 2013). Within Clade V, the tetraploid species *B. alnoides* and *B. cylindrostachya* form an unresolved cluster with the two diploid species, *B. luminifera* and *B. hainanensis*, indicating their common ancestry (Fig. 1).

*Betula nigra* is placed outside the subgenus *Betula* in all of our ITS phylogenies, both with and without unverified samples, and with and without polyploids in the analyses (Figs 1 and 2; Supplementary Data Fig. S2). In contrast, a phylogenetic study based on *NIA* suggests that it is more closely related to species of subgenus *Betula* than *B. alnoides* (Li *et al.*, 2007), and morphologically *B. nigra* is most similar to *B. dahurica* (subgenus *Betula*). The phylogenetic position of *B. nigra* needs further research based on multiple loci.

### Genome size and ploidy evolution

Different ploidy levels are present in all subgenera and sections of *Betula* except subgenus *Nipponobetula*, indicating several independent occurrences of polyploidy in the evolution of the genus (Järvinen *et al.*, 2004). Only subgenus *Aspera* contains ploidy levels above octoploid (Fig. 3; Supplementary Data Table S1).

The narrow ranges of these species of subgenus *Aspera* with high ploidy level (e.g. *B. insignis*, *B. megrelica*, *B. globispica* and *B. fargesii*) may indicate that they are of recent origin or have low invasiveness perhaps due to a low growth rate, which has been associated with larger genome size (Lavergne *et al.*, 2010; Fridley and Craddock, 2015), or their lack of, or very narrow, seed wings (Ashburner and McAllister, 2013). The narrow distributions of these relatively large genomes may also be influenced by available nutrients, such as nitrogen or phosphorus which may select against plants with large genome sizes (Knight *et al.*, 2005; Leitch and Leitch, 2012), and low temperature, which may influence the rate of cell division (Grime and Mowforth, 1982). On the other hand, these high ploidy level birches occur in areas known to harbour many relictual species, and their small populations may be relicts from larger distributions in the past. In contrast, the most diversified, widespread and ‘successful’ species are members of subgenus *Betula* with low ploidy levels (such as *B. pendula*, *B. nana* and *B. glandulosa*). Hybridization and adaptive introgression occur frequently within subgenus *Betula* (Thórsson *et al.*, 2010), which may play an important role in colonization of new habitats.

Our genome size results agree with published genome sizes for Icelandic birches, *B. nana* and *B. pubescens*, which suggest that no significant genome downsizing has occurred in tetraploid *B. pubescens* (Anamthawat-Jónsson *et al.*, 2010). However, our results for the 2C-value of *B. populifolia* are over twice as large as those measured by Feulgen microdensitometry (Olszewska and Osiecka, 1984). This is unlikely to be simply due to the difference in methodology, as flow cytometry and Feulgen microdensitometry were shown to give congruent measurements for Icelandic birches (Anamthawat-Jónsson *et al.*, 2010). Specimen misidentification is also unlikely to be the cause of the differences, as all of the *Betula* species that we measured have a 2C-value of more than twice the measure of the 2C-value of *B. populifolia* (Olszewska and Osiecka, 1984); perhaps chemical interference (Greilhuber, 2008) is the explanation for their unusual result. We also found the previously reported 2C-value of *B. nigra* at 2·90 pg (Bai *et al.*, 2012) to be large compared with the 2C-value of 0·88 pg for *B. nigra* here, and the specimen measured by Bai *et al.* (2012) has now been identified as *B. alleghaniensis* through checking the voucher specimen (DOB0420) (Professor Waller, pers. comm.), which is congruent with the 2C-value of 2·97 pg of *B. alleghaniensis* found here (Supplementary Data Table S1).

We found the monoploid genome size (1Cx-value) for most species of *Betula* to be between 0·42 pg and 0·57 pg. Four outlier species, two with lower 1Cx-values and two with higher 1Cx-values, all have higher ploidy levels: octoploid *B. murrayana* (1Cx = 0·38 pg), octoploid *B. chinensis* (1Cx = 0·39 pg), hexaploid *B. dahurica* (1Cx = 0·60 pg) and octoploid *B. dahurica* (1Cx = 0·57 pg). The chromosome counts of these accessions need to be double-checked, but, assuming they are correct, we found a general pattern that the variance of 1Cx genome sizes is greater in the species of *Betula* with higher ploidy levels than it is in the diploid species. This suggests that upsizing or downsizing of the sizes of the genomes is occurring in the polyploid birches, perhaps through loss of genome fragments (Buggs *et al.*, 2009, 2012), or proliferation of transposable elements (Bennetzen *et al.*, 2005).

### Biogeography

The phylogeography of several species of *Betula* has been extensively studied. In general, widespread species, such as *B. pubescens*/*B. pendula* (Maliouchenko *et al.*, 2007) in Europe and *B. papyrifera*/*B. alleghaniensis* in North America (Thomson *et al.*, 2015) show little population subdivision even at large scale, perhaps due to rapid population growth and high levels of gene flow, due to dispersal of pollen and seeds over long distances. In contrast, species likely to have lower dispersal ability, such as *B. nana* (Wang *et al.*, 2014*a*), *B. humilis* (Jadwiszczak *et al.*, 2012) and *B. maximowicziana* (Tsuda and Ide, 2005; Tsuda *et al.*, 2015), reveal a more subdivided genetic population structure. In addition, geographic barriers in the past and present may play an important role in causing genetic discontinuity (Eidesen *et al.*, 2013).

To our knowledge, biogeographical disjunctions among *Betula* species have only been mentioned in Li *et al.* (2005), based on a smaller sample size. Species of Clade III have disjunct distributions (Ashburner and McAllister 2013), with *B. medwediewii* and *B. megrelica* in Georgia and Turkey, and *B. lenta* in North America. We speculate that their common ancestor may have been continuously distributed over the northern hemisphere. Subsequent climate change may have eliminated it in intervening regions, causing geographical disjunctions. In addition, this genus contains three groups with disjunct distributions between North-east Asia and South-west Asia: a common disjunction in groups of related species (Ran *et al.*, 2006). Within subsection *Asperae*, *B. schmidtii* and *B. chichibuensis* occur in North-east Asia whereas *B. calcicola*, *B. potaninii* and *B. delavayi* occur only in South-west China. In the clade comprising subsection *Chinenses*, *B. globispica* occurs in North-east Asia, whereas *B. fargesii* occurs in South-west and central China. In the clade comprising *B. costata*, *B. utilis* and *B. ashburneri* (section *Costatae*), the first species occurs in North-east Asia whereas the latter two are in South-west and central China.

### Unexpected phylogenetic positions of unverified accessions

Unexpected phylogenetic signals for a subset of taxa in our phylogeny of all samples led us to re-appraise their identification. The *B. fruticosa* and *B. nana* ssp. *exilis* (synonym *B. glandulosa*) samples from Helsinki Botanic Garden were determined to be a subspecies of *B. pendula* and *B. pumila*, respectively, based on ITS and morphology (examined by H.A.M.). The putative *B. skvortsovii* sample was determined to be *B. ashburneri* based on ITS, morphology (examined by H.A.M.) and genome size of 1·00 pg (2C-value). The nesting of two accessions of *B. glandulosa* into a clade including *B. pumila*, whereas the verified *B. glandulosa* was placed into a distinct clade, was probably caused by the misidentification of *B. pumila* as *B. glandulosa* due to their morphological similarity (Fig. 2). Similarly, *B. pendula* is sometimes misidentified as *B. pubescens*, and vice versa, as there is a continuum of leaf variations between the two (Wang *et al.*, 2014*b*).

In addition, of the 12 sequences downloaded from GenBank, we think that at least five were possibly misidentified: *B. costata* (AY352337.1), *B. insignis* (KP092744.1), *B. glandulosa* (AY761110.1), *B. dahurica* (FI011773) and *B. chinensis* (AY761105.1). The fact that *B. dahurica* (FI011773) was collected from the Himalaya region is a strong signal of its misidentification because *B. dahurica* is distributed in North-east Asia. This species is more likely to be *B. utilis* as *B. utilis* is common in the Himalaya region, and this fits with the ITS data. There are 12 accessions clustered with a clade of *B. pubescens*/*B. pendula*, showing unexpected phylogenetic signals (Fig. 2). Besides the one labelled as *B. fruticosa* that is a clear misidentification, the remaining unexpected placements may be caused by hybridization or gene flow between *B. pubescens*/*B. pendula*, as these species (such as *B. nana*, *B. glandulosa*, *B. humilis*, *B. occidentalis*, *B. turkstanica* and *B. papyrifera*) can hybridize naturally or in cultivation with *B. pubescens*/*B. pendula* (Barnes *et al.*, 1974; Sulkinoja, 1990; Truong *et al.*, 2007; Jadwiszczak *et al.*, 2012; Ashburner and McAllister, 2013).

### Concluding remarks

Phylogenentic analyses of the genus *Betula* based on ITS sequences provide broad agreement with Ashburner and McAllister’s (2013) taxonomical treatment of this genus. This study gives us some new information about the possible origins of some polyploids in the genus, such as *B. alnoides*, *B. chinensis*, *B. delavayi*, *B. medwediewii* and *B. megrelica*, but the origins of *B. bomiensis* and *B. grossa* remain ambiguous. The phylogenetic positions of *B. michauxii*, *B. maximowicziana* and *B. nigra* remain questionable. The phylogenetic relationships of the genus *Betula* needs to be further addressed using multiple loci and next-generation sequencing methods such as restriction site-associated DNA markers, which have been successfully applied to *Betula* species in a pilot study (Wang *et al.*, 2013).

## SUPPLEMENTARY DATA

Supplementary data are available online at www.aob.oxfordjournals.org and consist of the following. Table S1: detailed information of the taxa used for ITS sequencing and taxa used for genome size estimation. Table S2: detailed information of the taxa used for comparing the average ploidy level and the mean 2C value of genome size of different ranges. Figure S1: Bayesian analysis of verified *Betula* species using ITS sequences. Figure S2: phylogenetic tree from the maximum likelihood analysis of *Betula* diploids using ITS.

Supplementary Data
