# Supplementary material for: Molecular phylogeny and genome size evolution of the genus Betula (Betulaceae)
Source: Ann Bot. 2016 Apr 11;117(6):1023–35. doi: 10.1093/aob/mcw048 (PMC4866320; doi:10.1093/aob/mcw048)
Supplement: Supplementary Data [file supp_117_6_1023__index.html]

Molecular phylogeny and genome size evolution of the genus Betula (Betulaceae) — Supplementary Data 

# Molecular phylogeny and genome size evolution of the genus *Betula* (Betulaceae)

## Supplementary Data

files

- Supplementary Data - docx file
