# Supplementary material for: Molecular phylogeny and genome size evolution of the genus Betula (Betulaceae)
Source: Ann Bot. 2016 Apr 11;117(6):1023–35. doi: 10.1093/aob/mcw048 (PMC4866320; doi:10.1093/aob/mcw048)

**Online Supplementary Information**

**Molecular phylogeny and genome size evolution of the genus *Betula* (Betulaceae)**

Nian Wang, Hugh A. McAllister, Paul R. Bartlett, Richard J. A. Buggs*

**Table S1** Detailed information of the taxa used for ITS sequencing and taxa used for genome size estimation.

**Table S2.** Detailed information of the taxa used for comparing the average ploidy level and the mean 2C value of genome size of different ranges.

**Figure S1** Bayesian analysis of verified *Betula* species using ITS sequences. Species were classified according to Ashburner and McAllister (2013). Values are posterior probabilities above 0.54.

**Figure S2** Phylogenetic tree from the maximum likelihood analysis of *Betula* diploids (with an identity checked by H. McAllister) using ITS. Species were classified according to Ashburner and McAllister (2013). Values above branches are bootstrap percentages of ≥ 50%.

**Table S1. Detailed information of the taxa used for ITS sequencing and genome size estimation.**

| Species^1^ | Genome size^2^  (s.d.)/pg | Ploidy level | Living collection^3^ | Native range | GenBank accession number^4^ | Herbarium accession numbers^5^ |
| --- | --- | --- | --- | --- | --- | --- |
| ***B. albosinensis* Burkill** |  |  | H |  | KT308924 | BM001122956 |
| *B. albosinensis* Burkill | 2.06 (0.04) | 4 | N | China | KT308954 | BM001123036 |
| *B. albosinensis* Burkill var. *septentrionalis* C. K. Schneider | 2.04 (0) | 4 | SL | Sichuan, China | KT308947 | BM001122996 |
| *B. alleghaniensis* Britton | 2.97 (0.01) | 6 | N | Tenessee, USA | KT308925 | BM001123048 |
| *B. alnoides* Buchanan-Hamilton ex D. Don | 1.95 (0.01) | 4 | n/a | Guangxi, China | KT308940 | BM001122936 |
| *B. apoiensis* Nakai ex H.Hara | 2.07 (0.01) | 4 | SL |  | n/a | BM001123007 |
| *B. ashburneri* McAllister & Rushforth |  |  | RBGE | Bhutan | KT308952 | E 19841878 A |
| *B. ashburneri* McAllister & Rushforth | 0.98 (0) | 2 | SL | SE Tibet, China | KT308953 | BM001122997 |
| *B. ashburneri* McAllister & Rushforth | 0.99 (0) | 2 | SL | Shanxi, China | n/a | BM001122998 |
| *B. ashburneri* McAllister & Rushforth | 0.98 (0.01) | 2 | SL | Nepal | n/a | BM001123006 |
| *B. bomiensis* P.C.Li |  |  | RBGE | Tajikistan | KT308912 | E 20110653 A |
| *B. bomiensis* P.C.Li | 2.20 (0) | 4 | N | Tibet, China | KT308911 | BM001123011 |
| ***B. browicziana* Güner** |  |  | RBGE | Turkey | KT308968 | E 20081535 C |
| *B. calcicola* (W.W.Sm.) P.C.Li | 0.91 (0.01) | 2 | N | Yunnan, China | KT308914 | BM001123012 |
| *B. chichibuensis* Hara | 0.92 (0.01) | 2 | SL | Japan | KT308916 | BM001122959 |
| *B. chichibuensis* Hara | 0.91 (NA) | 2 | SL | Japan | KT308915 | BM001122958 |
| *B. chinensis* Maxim. | 2.76 (0.01) | 6 | N | S. Korea | KT308917 | BM001123013 |
| *B. chinensis* Maxim. | 3.12 (0.03) | 8 | N | S. Korea | KT308918 | BM001123014 |
| *B. cordifolia* Regel |  |  | RBGE | USA | KT309015 | E 19961304 A |
| *B. cordifolia* Regel | 0.96 (0) | 2 | N | Canada | KT309016 | BM001123015 |
| *B. cordifolia* Regel | 1.00 (0) | 2 | SL | Canada | n/a | BM001122960 |
| *B. corylifolia* Regel & Maxim |  |  | RBGE | Japan | KT308907 | E 20052047 P |
| *B. corylifolia* Regel & Maxim | 0.97 (0) | 2 | RBGE | Japan | KT308908 | E 20052047 O |
| *B. costata* Trautv. | 0.93 (0) | 2 | N | Beijing, China | KT308958 | BM001123016 |
| *B. cylindrostachya* Lindl. ex Wall | 1.91 (0.01) | 4 | SL | India | KT308941 | BM001122961 |
| *B. dahurica* Pall. | 4.57 (0) | 8 | N | Hokkaido, Japan | KT308963 | BM001123017 |
| *B. dahurica* Pall. | 3.60 (0.02) | 6 | SL | Hokkaido, Japan | KT308962 | BM001122962 |
| *B. dahurica* Pall. | 3.79 (0.02) | 6 | N | Russian Far East | n/a | BM001123039 |
| *B. dahurica* Pall. | 4.36 (0.04) | 8 | N | S. Korea | n/a | BM001123040 |
| *B. dahurica* Pall. | 4.48 (0.02) | 8 | N | Russian Far East | n/a | BM001123041 |
| *B. dahurica* Pall. | 4.53 (0.02) | 8 | N | Nobeyama, Japan | n/a | BM001123042 |
| *B. dahurica* Pall. | 4.45 (0.01) | 8 | N | Russian Far East | n/a | BM001123043 |
| *B. delavayi* Franch. |  |  | n/a | Yunnan, China | KT308913 | BM001122938 |
| ***B. delavayi* Franch.** |  |  | K |  | KT308922 |  |
| *B. delavayi* Franch. | 3.20 (0.01) | 6 | SL | Yunnan, China | KT308921 | BM001122963 |
| *B. ermanii* Cham. | 2.00 (0.01) | 4 | SL | Hokkaido, Japan | KT308956 | BM001122964 |
| *B. ermanii* Cham. |  |  | K |  | KT308957 |  |
| *B. ermanii* var. *lanata* Regel |  |  | H | Russia | KT308959 | BM001122957 |
| *B. ermanii* var. *lanata* Regel | 2.12 (0) | 4 | N | Russian Far East | KT30860 | BM001123018 |
| *B. fargesii* (Franchet) P. C. Li. | 5.17 (0.01) | 10 | N | Hubei, China | KT308906 | BM001123019 |
| ***B. fruticosa*** **Pall.** |  |  | H | Russia | KT309002 | BM001122955 |
| *B. glandulosa* Michaux |  |  | n/a | Canada | KT309017 | BM001122942 |
| ***B. glandulosa* Michaux** |  |  | K | Irkutsk, Russia | KT308995 |  |
| *B. globispica* Shirai |  |  | K |  | KT308904 |  |
| *B. globispica* Shirai | 4.88 (0.03) | 10 | N | Japan | KT308905 | BM001123020 |
| *B. grossa* Siebold & Zucc. |  |  | K | Honshu, Japan | KT308935 |  |
| *B. grossa* Siebold & Zucc. | 2.58 (0) | 6 | SL | Honshu, Japan | KT308934 | BM001122965 |
| *B. hainanensis* J. Zeng, B.Q. Ren, J.Y. Zhu & Z.D. Chen | 0.91 (0) | 2 | n/a | Hainan, China | KT308942 | BM001122937 |
| *B. halophila* Ching |  |  | n/a | Xinjiang, China | KT308967 |  |
| *B. humilis* Schrank |  |  | K |  | KT309025 |  |
| *B. humilis* Schrank | 0.98 (0.01) | 2 | N | Romania | KT309026 | BM001123021 |
| *B. humilis* Schrank | 0.94 (0.01) | 2 | SL | Poland | KT309024 | BM001122966 |
| *B. insignis* Franch. | 4.71 (0.01) | 10 | SL | Yunnan, China | KT308927 | BM001122967 |
| *B. insignis* Franch. |  |  | RBGE | Guizhou, China | KT308928 | E 20050415 R |
| *B. insignis* Franch. | 4.44 (0.02) | 10 | n/a | Guizhou, China | n/a | BM001122940 |
| *B. insignis* ssp. *fansipanensis* Ashburner & McAll. | 5.33 (0.01) | 10 | n/a | Yunnan, China | KT308929 | BM001122941 |
| *B. lenta* f. *uber* (Ashe) Fernald |  |  | K |  | KT308937 |  |
| *B. lenta* f. *uber* (Ashe) Fernald | 0.96 (0) | 2 | SL | Virginia, USA | KT308938 | BM001122994 |
| *B. lenta* L. | 0.95 (0) | 2 | SL | Vermont, USA | KT308936 | BM001122993 |
| *B. luminifera* H.Winkl. |  |  | K | Sichuan, China | KT308939 |  |
| *B. luminifera* H.Winkl. |  |  | N | Sichuan, China | KT308943 | BM001123047 |
| *B. luminifera* H.Winkl. | 1.00 (0.01) | 2 | RBGE | Yunnan, China | KT308944 | E 19933472 G |
| *B. maximowicziana* Regel | 0.93 (0) | 2 | SL | Japan | KT308945 | BM001122968 |
| *B. maximowicziana* Regel | 0.96 (0) | 2 | N | Japan | KT308946 | BM001123022 |
| *B. medwediewii* Regel |  |  | K |  | KT308930 |  |
| *B. medwediewii* Regel | 4.73 (0.02) | 10 | SL | Caucasus, Georgia | KT308931 | BM001123004 |
| *B. medwediewii* Regel | 4.78 (0.02) | 10 | N | Caucasus, Georgia | n/a | BM001123023 |
| *B. megrelica* D. Sosn. |  |  | K |  | KT308932 |  |
| *B. megrelica* D. Sosn. | 5.12 (0.01) | 12 | SL | Caucasus, Georgia | KT308933 | BM001122969 |
| *B. michauxii* Spach | 0.95 (0) | 2 | N | Canada | KT308978 | BM001123024 |
| *B. microphylla* Bunge | 1.81 (0) | 4 | N | Mongolia | KT308984 | BM001123025 |
| *B. middendorffii* Trautv. & C.A.Mey | 2.06 (NA) | 4 | n/a | Russian Far East | KT308986 | BM001123049 |
| *B. murrayana* B. V. Barnes & Dancik | 3.03 (0.01) | 8 | N | Ontario, Canada | KT308926 | BM001123026 |
| *B. nana* L. | 1.00 (0) | 2 | n/a | Scotland | KT309018 | BM001122943 |
| *B. nana* L. | 0.92 (0.01) | 2 | n/a | Scotland | KT309020 | BM001074532 |
| ***B. nana* ssp. *exilis* (Sukaczev) Hultén** |  |  | H | Canada | KT309019 | BM001122954 |
| *B. nigra* L. |  |  | K |  | KT308965 |  |
| *B. nigra* L. | 0.88 (0) | 2 | SL | USA | KT308964 | BM001122970 |
| ***B. obscura*** **Kotula** |  |  | K |  | KT308993 |  |
| *B. occidentalis* Hooker |  |  | H | Albert, Canada | KT309028 |  |
| *B. occidentalis* Hooker | 0.96 (0) | 2 | SL | Montana, USA | KT309027 | BM001122971 |
| *B. ovalifolia* Ruprecht |  |  | K |  | KT309023 |  |
| *B. ovalifolia* Ruprecht | 1.92 (0.01) | 4 | SL | Mongolia | KT309022 | BM001122972 |
| *B. papyrifera* Marshall |  |  | K |  | KT309012 |  |
| ***B. papyrifera* Marshall** |  |  | K |  | KT309013 |  |
| *B. papyrifera* Marshall | 2.94 (0.01) | 6 | SL | Ontarioo, Canada | KT309011 | BM001122973 |
| *B. papyrifera* Marshall | 2.94 (0.02) | 6 | SL | Minnosota, USA | n/a | BM001122974 |
| *B. papyrifera* Marshall var. *commutata* Regel | 2.95 (0.02) | 6 | SL | Vancouver, Canada | KT309014 | BM001122975 |
| *B. pendula* Roth ssp. *mandshurica* (Reg.) Nakai |  |  | H | Russia | KT308990 | BM001122952 |
| *B. pendula* Roth ssp. *mandshurica* (Reg.) Nakai |  |  | H | Milkovo, Bulgaria | KT308999 | BM001122953 |
| *B. pendula* Roth ssp. *mandshurica* (Reg.) Nakai | 0.95 (0) | 2 | N | Japan | KT308996 | BM001123028 |
| *B. pendula* Roth ssp. *mandshurica* (Reg.) Nakai | 0.98 (0.01) | 2 | N | Russian Far East | n/a | BM001123050 |
| *B. pendula* Roth ssp. *mandshurica* (Reg.) Nakai | 0.94 (0) | 2 | N | Alberta, Canada | KT309005 | BM001123029 |
| *B. pendula* Roth ssp. *mandshurica* (Reg.) Nakai | 0.93 (0.01) | 2 | SL | Hokkaido, Japan | KT309008 | BM001122976 |
| *B. pendula* Roth ssp. *pendula* Roth |  |  | n/a | England | KT309006 | BM001122944 |
| *B. pendula* Roth ssp. *pendula* Roth |  |  | H | Finland | KT309001 | BM001122950 |
| *B. pendula* Roth ssp. *pendula* Roth |  |  | H |  | KT309007 | BM001122951 |
| *B. pendula* Roth ssp. *pendula* Roth | 0.92 (0) | 2 | SL | Sicily | KT309000 | BM001122977 |
| *B. pendula* Roth ssp. *pendula* Roth | 0.91 (0) | 2 | SL | Poland | KT308997 | BM001122978 |
| *B. pendula* Roth ssp. *szechuanica* Ashburner & McAll. |  |  | K | Yunnan, China | KT308998 |  |
| *B. pendula* Roth ssp. *szechuanica* Ashburner & McAll. |  |  | K |  | KT309003 |  |
| *B. pendula* Roth ssp. *szechuanica* Ashburner & McAll. | 0.93 (0) | 2 | N | Sichuan, China | n/a | BM001123027 |
| *B. pendula* Roth ssp. *szechuanica* Ashburner & McAll. | 0.91 (0) | 2 | SL | Sichuan, China | KT309004 | BM001122979 |
| *B. pendula* Roth ssp. *szechuanica* Ashburner & McAll. | 0.99 (0.01) | 2 | SL | Sichuan, China | n/a | BM001122980 |
| *B. populifolia* Marshall |  |  | K |  | KT308994 |  |
| *B. populifolia* Marshall | 0.96 (0) | 2 | SL | Vermont, USA | KT309009 | BM001122981 |
| *B. populifolia* Marshall | 0.94 (0) | 2 | SL | Vermont, USA | KT309010 | BM001122982 |
| *B. potaninii* Batalin |  |  | K |  | KT308910 |  |
| *B. potaninii* Batalin | 1.08 (0) | 2 | N | Sichuan, China | KT308909 | BM001123030 |
| *B. pubescens* Ehrh. var. *celtiberica* Rivas Mart. |  |  | K |  | KT308972 |  |
| *B. pubescens* Ehrh. var. *celtiberica* Rivas Mart. | 1.88 (0.01) | 4 | SL | Spain | KT308977 | BM001122983 |
| *B. pubescens* Ehrh. var. *fragrans* Ashburner & McAll. | 1.88 (0.01) | 4 | SL | Scotland | KT308975 | BM001122985 |
| *B. pubescens* Ehrh. var. *fragrans* Ashburner & McAll. | 1.88 (0) | 4 | SL | Oslo, Norway | n/a | BM001122986 |
| *B. pubescens* Ehrh. var. *fragrans* Ashburner & McAll. | 1.94 (0) | 4 | N | Scotland | KT308974 | BM001123031 |
| *B. pubescens* Ehrh. var. *litiwinowii* Ashburner & McAll. | 1.84 (0.01) | 4 | N | Armenia | KT308983 | BM001123032 |
| *B. pubescens* Ehrh. var. *litiwinowii* Ashburner & McAll. | 1.79 (0.01) | 4 | SL | Caucasus, Georgia | KT308971 | BM001122987 |
| *B.pubescens* Ehrh. var. *murithii* (Gaudin ex Regel) Gremli | 1.87 (0) | 4 | SL | Switzerland | n/a | BM001123010 |
| *B. pubescens* Ehrh. var. *pubescens* |  |  | H | Czech Republic | KT308982 | BM001122948 |
| *B. pubescens* Ehrh. var. *pubescens* | 1.90 (0.01) | 4 | N | Turkey | n/a | BM001123046 |
| *B. pubescens* Ehrh. var. *pubescens* | 1.90 (0) | 4 | SL | NE Turkey | KT308981 | BM001122988 |
| *B. pubescens* Ehrh. var. *pubescens* |  |  | n/a | England | KT308969 | BM001122945 |
| *B. pubescens* Ehrh. var. *pubescens* |  |  | n/a | England | KT308970 | BM001122946 |
| *B. pubescens* Ehrh. var. *pubescens* | 1.91 (0.01) | 4 | n/a | England | n/a | BM001122947 |
| *B. pubescens* Ehrh. var. *pumila* (L.) Govaerts |  |  | H | Finland | KT308980 | BM001122949 |
| *B. pubescens* Ehrh. var. *pumila* (L.) Govaerts | 1.91 (0.01) | 4 | SL | Trondelog, Norway | KT308976 | BM001122989 |
| *B. pubescens* Ehrh. var. *pumila* (L.) Govaerts | 1.92 (0.02) | 4 | SL | Trondelog, Norway | n/a | BM001122990 |
| *B. pubescens* Ehrh. var. *pumila* (L.) Govaerts | 2.01 (0.01) | 4 | N | Norway | KT308973 | BM001123033 |
| *B. pumila* L. | 2.10 (0.01) | 4 | SL | Canada | KT309021 | BM001122991 |
| *B. raddeana* Trautv. | 2.84 (0) | 6 | SL | Georgia | KT308966 | BM001122992 |
| ***B. resinifera*** **Britton** |  |  | K | USA | KT308991 |  |
| *B. schmidtii* Regel |  |  | K |  | KT308919 |  |
| *B. schmidtii* Regel | 0.92 (0) | 2 | N | Russian Far East | KT308920 | BM001123034 |
| ***B. skvortsovii* McAll. & Ashburner** | 1.00 (0) | 2 | n/a |  | KT308961 |  |
| *B. tianshanica* Rupr. | 1.90 (NA) | 4 | RBGE | China | KT308989 | E 20051397 A |
| ***B. turkstanica*** **Litv.** |  |  | K | Tajikistan | KT308992 |  |
| *B. utilis* D. Don var. *jacquemontii* (Spach) Winkle | 2.09 (0) | 4 | SL | Nepal | KT308951 | BM001122995 |
| *B. utilis* D. Don var. *jacquemontii* (Spach) Winkle ‘Graywood Ghost’ | 2.16 (0.01) | 4 | SL | cultivar | n/a |  |
| *B. utilis* D. Don var. *jacquemontii* (Spach) Winkle ‘Gregory’ | 2.13 (0.02) | 4 | SL | cultivar | n/a |  |
| *B. utilis* D. Don var. *jacquemontii* (Spach) Winkle ‘Hergest’ | 1.98 (0) | 4 | SL | cultivar | n/a |  |
| *B. utilis* D. Don var. *jacquemontii* (Spach) Winkle ‘Knight’ | 2.07 (0.01) | 4 | SL | cultivar | n/a |  |
| *B. utilis* D. Don var. *jacquemontii* (Spach) Winkle ‘Doorenbos’ | 1.89 (0.01) | 4 | SL | cultivar | n/a |  |
| *B. utilis* D. Don var. *jacquemontii* (Spach) Winkle ‘Long Trunk’ | 1.98 (0.01) | 4 | SL | cultivar | n/a |  |
| *B. utilis* D. Don var. *jacquemontii* (Spach) Winkle ‘Sauwola white’ | 1.93 (0.02) | 4 | SL | cultivar | n/a |  |
| *B. utilis* D.Don | 2.12 (0) | 4 | N | Nepal | KT308948 | BM001123035 |
| *B. utilis* D.Don | 2.15 (0) | 4 | SL | Sichuan, China | KT308949 | BM001123005 |
| *B. utilis* D.Don var. *occidentalis* Ashburner & A.D.Schill. |  |  | K |  | KT308950 |  |
| ***B. utilis* D.Don var. *occidentalis* Ashburner & A.D.Schill.** | 1.78 (NA) | 4 | RBGE | Tajikistan | KT308923 | E 20110849 A |
| *B. utilis* D.Don var. *prattii* Burkill |  |  | K |  | KT308955 |  |
| *B. utilis* D.Don var. prattii Burkill | 2.10 (0.01) | 4 | N | SW China | n/a | BM001123044 |
| *B. x caerulea* Blanch. |  |  | K |  | KT308987 |  |
| *B. x caerulea* Blanch. | 0.97 (0) | 2 | SL | Vermont, USA | KT308988 | BM001122999 |
| *B. x minor* (Tuckerman) Fern. | 0.95 (0.01) | 2 | SL | Cananda | KT308985 | BM001123000 |
| *B. x utahensis* Britton | 1.82 (0.01) | 4 | SL | Montana, USA | KT308979 | BM001123001 |

^1^Bold font indicates taxa with unexpected phylogenetic positions and these taxa are listed in Fig.2 in brackets.  ^2^NA indicates less than three replicates were measured for genome size analysis; a blank means the genome size of this taxon was not estimated. ^3^SL: Stone Lane Gardens; N: Ness Gardens; K: Royal Botanic Gardens, Kew; RBGE: Royal Botanic Garden Edinburgh; H: Helsinki Botanic Garden in Finland; n/a: samples are not in a living collection. ^4^n/a: ITS not sequenced. ^5^Accession numbers starting with BM are for the Natural History Museum, London, and accession numbers starting E are for the Royal Botanic Garden, Edinburgh.

**Table S2. Detailed information of the taxa used for comparing the average ploidy level and the mean 2C value of the genome size of species different range sizes.**

| Species | 2C | 1C | 1x | Ploidy  level | Range^1^ | Section^2^ | Subgenus |
| --- | --- | --- | --- | --- | --- | --- | --- |
| *B. alnoides* Buchanan-Hamilton ex D. Don | 1.95 | 0.98 | 0.49 | 4 | M | *Acuminatae* | *Acuminata* |
| *B. cylindrostachya* Lindl. ex Wall | 1.91 | 0.96 | 0.48 | 4 | M | *Acuminatae* | *Acuminata* |
| *B. hainanensis* J. Zeng, B.Q. Ren, J.Y. Zhu & Z.D. Chen | 0.91 | 0.46 | 0.46 | 2 | M | *Acuminatae* | *Acuminata* |
| *B. luminifera* H.Winkl. | 1.00 | 0.50 | 0.50 | 2 | W | *Acuminatae* | *Acuminata* |
| *B. maximowicziana* Regel | 0.93 | 0.47 | 0.47 | 2 | M | *Acuminatae* | *Acuminata* |
| *B. bomiensis* P.C.Li | 2.20 | 1.10 | 0.55 | 4 | N | *Asperae* | *Aspera* |
| *B. calcicola* (W.W.Sm.) P.C.Li | 0.91 | 0.46 | 0.46 | 2 | N | *Asperae* | *Aspera* |
| *B. chichibuensis* Hara | 0.91 | 0.46 | 0.46 | 2 | M | *Asperae* | *Aspera* |
| *B. chinensis* Maxim. | 2.76 | 1.38 | 0.46 | 6 | M | *Asperae* | *Aspera* |
| *B. chinensis* Maxim. | 3.12 | 1.56 | 0.39 | 8 | M | *Asperae* | *Aspera* |
| *B. delavayi* Franch. | 3.20 | 1.60 | 0.53 | 6 | N | *Asperae* | *Aspera* |
| *B. fargesii* (Franchet) P. C. Li. | 5.17 | 2.59 | 0.52 | 10 | N | *Asperae* | *Aspera* |
| *B. globispica* Shirai | 4.88 | 2.44 | 0.49 | 10 | N | *Asperae* | *Aspera* |
| *B. potaninii* Batalin | 1.08 | 0.54 | 0.54 | 2 | N | *Asperae* | *Aspera* |
| *B. schmidtii* Regel | 0.92 | 0.46 | 0.46 | 2 | M | *Asperae* | *Aspera* |
| *B. alleghaniensis* Britton | 2.97 | 1.49 | 0.50 | 6 | M | *Lentae* | *Aspera* |
| *B. grossa* Siebold & Zucc. | 2.58 | 1.29 | 0.43 | 6 | M | *Lentae* | *Aspera* |
| *B. insignis* Franch. | 4.71 | 2.36 | 0.47 | 10 | M | *Lentae* | *Aspera* |
| *B. insignis* ssp. *fansipanensis* Ashburner & McAll. | 5.33 | 2.67 | 0.53 | 10 | N | *Lentae* | *Aspera* |
| *B. lenta* L. | 0.96 | 0.48 | 0.48 | 2 | M | *Lentae* | *Aspera* |
| *B. medwediewii* Regel | 4.73 | 2.37 | 0.47 | 10 | N | *Lentae* | *Aspera* |
| *B. megrelica* D. Sosn. | 5.12 | 2.56 | 0.43 | 12 | N | *Lentae* | *Aspera* |
| *B. murrayana* B. V. Barnes & Dancik | 3.03 | 1.52 | 0.38 | 8 | N | *Lentae* | *Aspera* |
| *B. humilis* Schrank | 0.94 | 0.47 | 0.47 | 2 | VW | *Apterocaryon* | *Betula* |
| *B. michauxii* Spach | 0.95 | 0.48 | 0.48 | 2 | M | *Apterocaryon* | *Betula* |
| *B. nana* L. | 1.00 | 0.50 | 0.50 | 2 | VW | *Apterocaryon* | *Betula* |
| *B. ovalifolia* Ruprecht | 1.92 | 0.96 | 0.48 | 4 | M | *Apterocaryon* | *Betula* |
| *B. pumila* L. | 2.10 | 1.05 | 0.53 | 4 | W | *Apterocaryon* | *Betula* |
| *B. cordifolia* Regel | 0.96 | 0.48 | 0.48 | 2 | W | *Betula* | *Betula* |
| *B. microphylla* Bunge | 1.81 | 0.91 | 0.45 | 4 | M | *Betula* | *Betula* |
| *B. occidentalis* Hooker | 0.96 | 0.48 | 0.48 | 2 | VW | *Betula* | *Betula* |
| *B. papyrifera* Marshall | 2.95 | 1.47 | 0.49 | 6 | VW | *Betula* | *Betula* |
| *B. pendula* Roth ssp. *pendula* Roth | 0.91 | 0.46 | 0.46 | 2 | VW | *Betula* | *Betula* |
| *B. populifolia* Marshall | 0.94 | 0.47 | 0.47 | 2 | W | *Betula* | *Betula* |
| *B. pubescens* Ehrh. var. *pubescens* | 1.88 | 0.95 | 0.48 | 4 | VW | *Betula* | *Betula* |
| *B. tianshanica* Rupr. | 1.90 | 0.95 | 0.48 | 4 | M | *Betula* | *Betula* |
| *B. ashburneri* McAllister & Rushforth | 0.99 | 0.50 | 0.50 | 2 | M | *Costatae* | *Betula* |
| *B. costata* Trautv. | 0.93 | 0.47 | 0.47 | 2 | M | *Costatae* | *Betula* |
| *B. ermanii* Cham. | 2.06 | 1.00 | 0.50 | 4 | W | *Costatae* | *Betula* |
| *B. utilis* D.Don | 2.08 | 1.02 | 0.51 | 4 | VW | *Costatae* | *Betula* |
| *B. dahurica* Pall. | 3.60 | 1.80 | 0.60 | 6 | M | *Dahuricae* | *Betula* |
| *B. dahurica* Pall. | 4.57 | 2.29 | 0.57 | 8 | M | *Dahuricae* | *Betula* |
| *B. nigra* L. | 0.88 | 0.44 | 0.44 | 2 | M | *Dahuricae* | *Betula* |
| *B. raddeana* Trautv. | 2.84 | 1.42 | 0.47 | 6 | N | *Dahuricae* | *Betula* |
| *B. corylifolia* Regel & Maxim | 0.97 | 0.49 | 0.49 | 2 | N | *Nipponobetula* | *Nipponobetula* |

^1^N, M, W and VW indicate narrow (species occuring in a single or a few localities and tend to be endangered), medium (species occuring commonly in multiple areas), widespread (species spread within some parts of a continent) and very widespread (species spread extensively within a continent or across continents) ranges, respectively.

^2^Species were classified according to Ashburner and McAllister (2013).

**Figure S1** Baysian analysis of verified *Betula* L. species using ITS sequences. Species were classified according to Ashburner and McAllister (2013). Values above branches show posterior probabilities above 0.54.


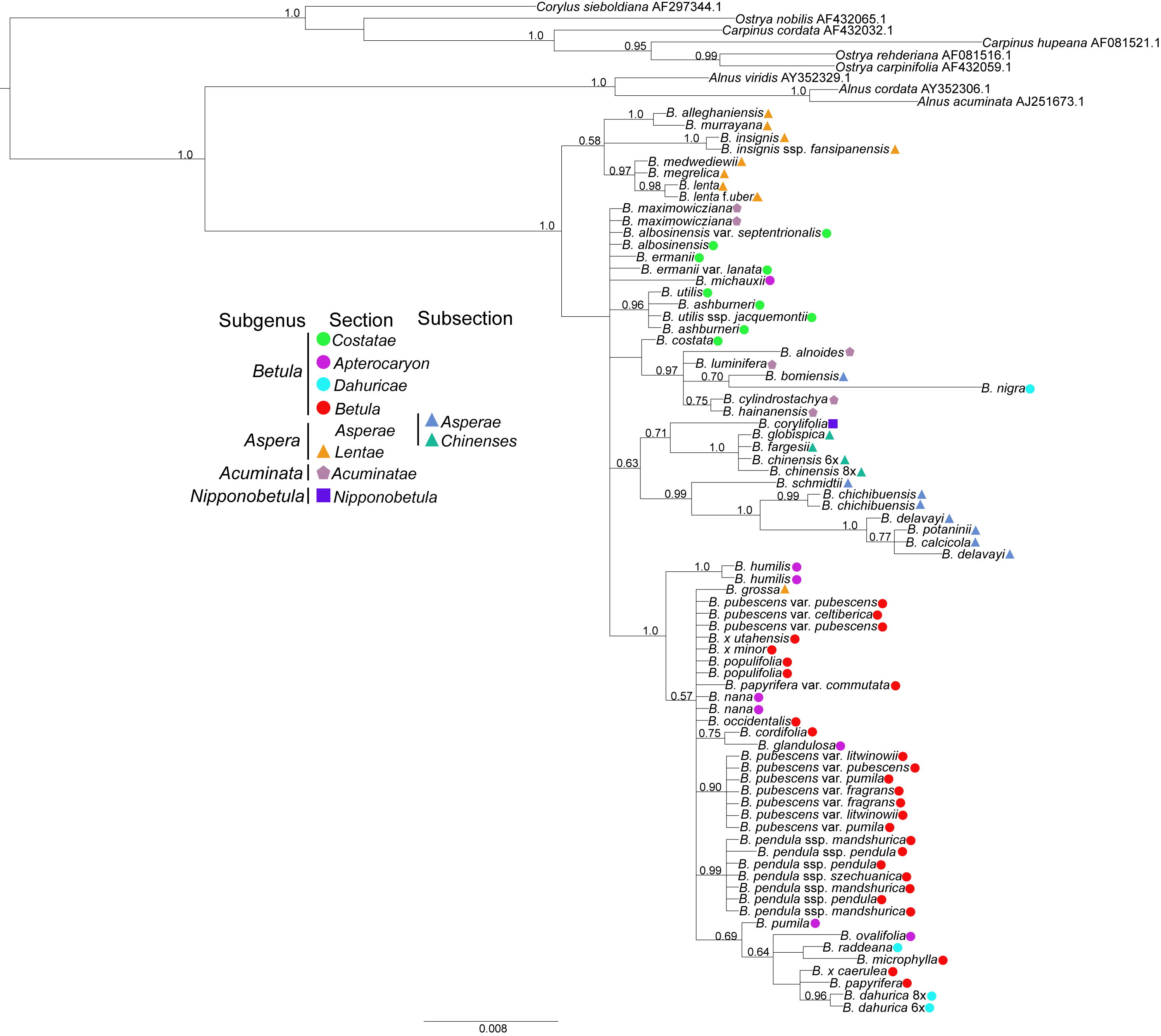


**Figure S2** Phylogenetic tree from maximum likelihood analysis of verified *Betula* L. diploids using ITS sequences. Species were classified according to Ashburner and McAllister (2013). Values above branches are bootstrap percentages of ≥ 50%.


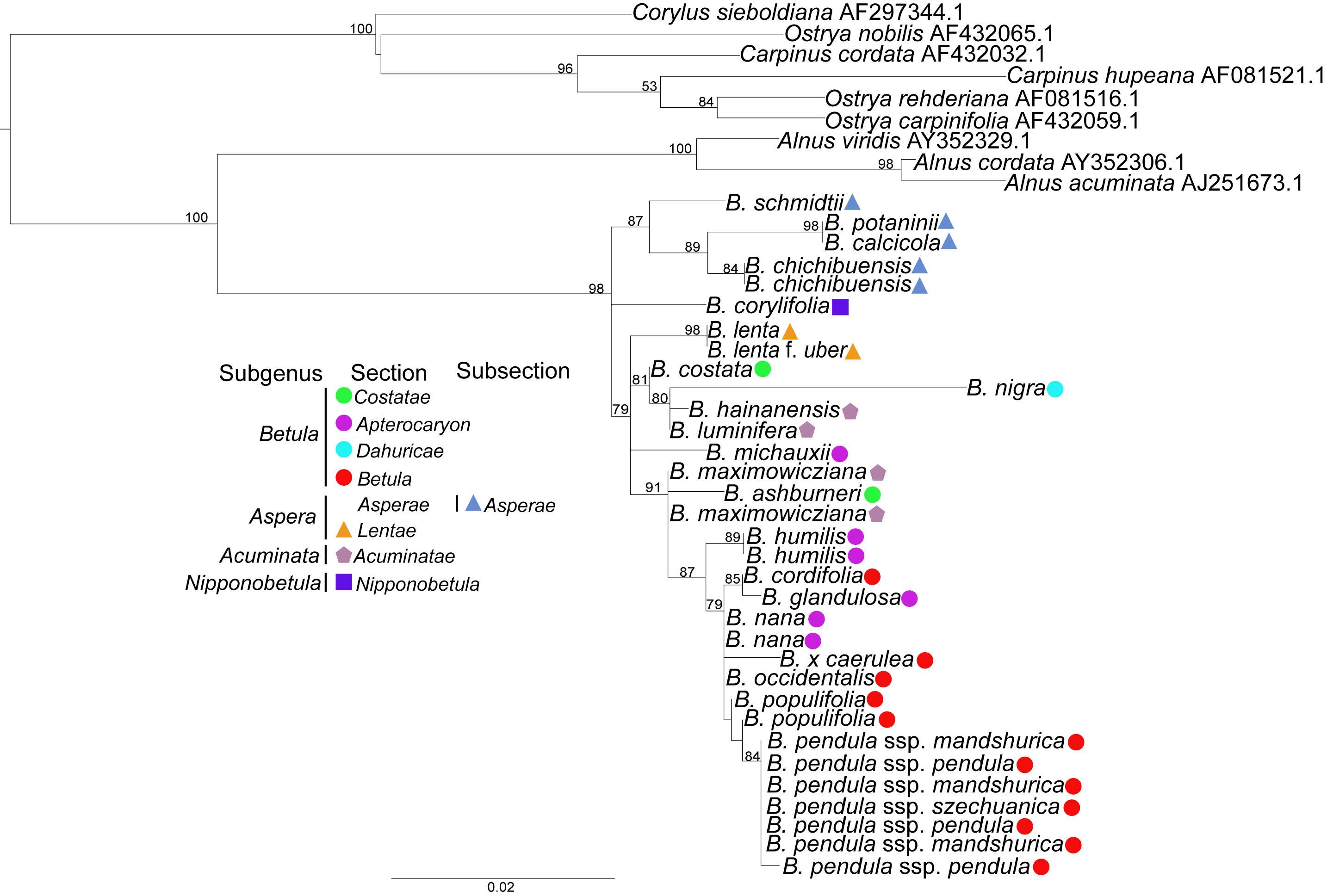

Supplement: Supplementary Data [file supp_mcw048_AnnBot_20160310_corrected.docx]
